# Impact of Storage Conditions on the Stability of Predominant Phenolic Constituents and Antioxidant Activity of Dried *Piper betle* Extracts

**DOI:** 10.3390/molecules23020484

**Published:** 2018-02-23

**Authors:** Ameena Ali, Chien Hwa Chong, Siau Hui Mah, Luqman Chuah Abdullah, Thomas Shean Yaw Choong, Bee Lin Chua

**Affiliations:** 1 School of Engineering, Taylor’s University, Lakeside Campus, No 1, Jalan Taylor’s, Subang Jaya, Selangor 47500, Malaysia; beelin.chua@taylors.edu.my; 2 School of Engineering and Physical Sciences, Heriot-Watt University, Malaysia Campus, No 1 Jalan Venna P5/2, Precinct 5, Putrajaya 62200, Malaysia; chien_hwa.chong@hw.ac.uk; 3 School of Biosciences, Taylor’s University, Lakeside Campus, No 1, Jalan Taylor’s, Subang Jaya, Selangor 47500, Malaysia; SiauHui.Mah@taylors.edu.my; 4 Department of Chemical and Environmental Engineering, University Putra Malaysia, UPM Serdang, Selangor 43400, Malaysia; chuah@upm.edu.my (L.C.A.); csthomas@upm.edu.my (T.S.Y.C.)

**Keywords:** *Piper betle*, storage, stability, total phenolic content, antioxidant capacity

## Abstract

The phenolic constituents in *Piper betle* are well known for their antioxidant potential; however, current literature has very little information on their stability under the influence of storage factors. Present study evaluated the stability of total phenolic content (TPC) and antioxidant activity together with individual phenolic constituents (hydroxychavicol, eugenol, isoeugenol and allylpyrocatechol 3,4-diacetate) present in dried *Piper betle*’s extract under different storage temperature of 5 and 25 °C with and without light for a period of six months. Both light and temperature significantly influenced TPC and its corresponding antioxidant activity over time. More than 95% TPC and antioxidant activity was retained at 5 °C in dark condition after 180 days of storage. Hydroxychavicol demonstrated the best stability with no degradation while eugenol and isoeugenol displayed moderate stability in low temperature (5 °C) and dark conditions. 4-allyl-1,2-diacetoxybenzene was the only compound that underwent complete degradation. A new compound, 2,4-di-*tert*-butylphenol, was detected after five weeks of storage only in the extracts exposed to light. Both zero-order and first-order kinetic models were adopted to describe the degradation kinetics of the extract’s antioxidant activity. Zero-order displayed better fit with higher correlation coefficients (*R*^2^ = 0.9046) and the half-life was determined as 62 days for the optimised storage conditions (5 °C in dark conditions).

## 1. Introduction

*Piper betle* is one of the most potent medicinal herbs that have been exploited over the years [1]. In addition to the vast number of beneficial properties, *Piper betle* has been also dubbed as a promising source of natural antioxidant agents in herbal healthcare products [2,3]. Studies indicate that the antioxidant activity of this herb is mainly due to the presence of the phenolic constituents, hydroxychavicol, eugenol and isoeugenol [4,5]. Hydroxychavicol has been reported to be a highly effective scavenger of reactive oxygen species (ROS) including peroxide, superoxide, and hydroxyl radicals [6]. Likewise, the radical scavenging activities of both eugenol and isoeugenol with ferric reducing capacities has also been reported previously [7]. All three phenolic compounds were found to inhibit lipid peroxidation and possessed protective activity against oxidative DNA damage [8,9]. The phenolics in *Piper betle* have shown great potential to be utilised as natural antioxidants in dietary and healthcare products.

The utilisation of these bioactive compounds from *Piper betle* in industrial applications sounds very promising, though their sensitive nature makes it tedious. Exposure to environmental changes such as light, humidity and temperature may lead to their degradation or even facilitate it [10]. Degradation of bioactive compounds is the prime origin for the loss of quality and nutrition of any herbal product for which it has been a topic of immense research for decades [11,12]. Koyu and Haznedaroglu reviewed the effect of light, temperature and humidity on the degradation of the major constituents present in dried extracts of the herb *Hypericum perforatum* L. (Hypericaceae) [13]. The authors concluded that all flavonoids underwent some form of degradation with exposure to the physio-chemical factors of environment. Chaaban et al. reported severe degradation of multiple phenolic flavonoids when exposed to light and oxygen, though the extent of degradation varied due to structural differences [12].

In the food and pharmaceutical industries, stability investigations are often sought after by manufacturers to study on degradation and its relative impact on the quality of herbal extracts or products containing herbal extracts [14]. In doing so, they protect their reputation by gaining approval from the regulatory bodies to reassure the customers of their product’s quality. Through stability investigations, the quality of the herbal extracts can be assessed in diverse environmental conditions. This enables to determine the proper storage condition under which the continuity of its properties can be ensured in the latter stages including its quality and safety, as well as its efficacy [15].

Degradation of the bioactive compounds can take place during multiple stages, including pre-treatment, processing and storage as medicinal herbs are subjected to drying, extraction, and long term storage [16,17]. Although, stability of the bioactive compounds present in *Piper betle* and their antioxidant potential during pre-treatment and processing has been previously reported, no stability study has been undertaken for storage parameters [4,18]. Therefore, the aim of this paper is to evaluate the impact of multiple environmental factors on the stability of dried *Piper betle*’s extract. This will be achieved by monitoring the impact of light and temperature on the total phenolic content and antioxidant capacity of the dried extract for a storage duration of 6 months. At the same time, the stability of the individual phenolic compounds (hydroxychavicol, eugenol and isoeugenol) in the dried extracts will be also monitored via gas chromatography–mass spectrometry (GC/MS) under the same storage conditions for the same duration. Lastly, the degradation kinetics of the antioxidant activity would be investigated by employing both zero-order and first-order kinetic models in order to determine the best model that describes the stability of *Piper betle* extracts.

## 2. Results

### 2.1. Effect of Storage Temperature and Light on the Stability of Retained Total Phenolic Content (TPC)

Storage temperature was found to impose a profound influence on TPC (Table 1) with variations observed throughout the entire storage period of 180 days. TPC of dried extract stored at low temperature of 5 °C with and without exposure to light showed the best stability with more than 99% retention. However, the TPC of the extracts stored at room temperature of 25 °C displayed slight reduction with retention of approximately 97% (dark) and 93% (light) respectively. In reference to the statistical analysis of multivariate analysis of variance (MANOVA), both temperature and light were found to have significant impact (*p* < 0.05) on TPC. Even though no severe changes were observed during the entire study, the minimal decrease in TPC, particularly for the samples stored in transparent bottles at 25 °C, could be attributed to the degradation of the phenolics present in the dried extracts. 

Multiple storage factors such as light, temperature, oxygen and pH have been highlighted to have affected the stability of the phenolic compounds [19,20]. Kotsiou and Tasioula-Margari reported reduction of more than 7% in the TPC of different varieties of olive oil at temperatures of 18 and 25 °C in the dark over storage period of 24 months. The authors claimed that the primary reason behind the degradation of phenolic compounds in the above temperatures were oxidation and hydrolysis [21]. The results clearly showed the immense effect of storage temperature on the stability of the phenolic compounds which can be facilitated in the presence of light. Del-Toro-Sánchez et al. reported a massive 36% reduction in the TPC of the herb, *Anemopsis californica*, extracts stored at 50, 25 and 4 °C with exposure to light after 180 days. The authors reported that high temperature and light degraded the structure of the polyphenols [22]. Qu et al. investigated the storage stability of phenolic compounds in sterilised liquid pomegranate peel extracts at 4 °C under light and dark conditions. The authors reported that major degradation and browning of ascorbic acid and polyphenols took place even at low temperatures due to oxidation and polymerisation reactions induced with exposure to light [23]. Corroborating with present study, similar oxidative reactions could have taken place at 25 °C that lead to the subsequent decrease in the TPC and exposure to light further facilitated its degradation.

### 2.2. Impact of Storage Conditions on Retained DPPH• Antioxidant Activity

In correspondence to the results of 2,2-diphenyl-1-picrylhydrazyl (DPPH•) antioxidant activity (Table 2), the influence of temperature was more profound than light. The importance of the two storage factors were further affirmed by MANOVA (Table S1) where both light and temperature was found to be statistically significant in influencing the antioxidant capacity of dried *Piper betle* extract. The best stability was observed at 5 °C in dark conditions which retained 99.98% of DPPH• antioxidant activity after 180 days of storage. On the contrary, both storage conditions of 5 °C (light) and 25 °C (dark) retained 96% of DPPH• antioxidant activity. The dried extracts at 25 °C with exposure to light were the least stable as only 90% of the DPPH• antioxidant activity was retained after 180 days.

Storage temperature and light has been found to be a major hindrance when it comes to the stability of phenolic compounds as well as their corresponding antioxidant activity. Zamora et al. reported significant decrease in the total antioxidant activity of 26 varieties of teas within three months of storage at room temperature of 25 °C [24]. The results of present study are also consistent with the findings of Zorić et al. who reported major decline in the antioxidant activity of Marasca sour cherry powder at 37 °C compared to the samples stored at 4 °C [25]. The authors further highlighted that the antioxidant capacity of the samples at 4 °C after 12 months were almost same to that of 37 °C after a mere three months, indicating faster degradation of the phenolic compounds at higher temperatures. Similarly in the present study, 95% of the antioxidant activity was retained after 180 storage days at 5 °C which is almost equivalent to the antioxidant activity of the samples at 25 °C after 75 days.

Primarily, the loss of the antioxidant activity could be attributed to the oxidation of the phenolic compounds as a result of being stored at high temperatures with exposure to light [24,26]. According to Gião et al., oxidation of the phenolic compounds in four different aromatic herbs consisting of sage, thyme, savory and sweet-amber lead to the reduction of their corresponding antioxidant activity by more than 30% during one year of storage at 25 °C in dark conditions [27]. Therefore, in the present study, Pearson’s correlation statistics was employed to establish the relationship between TPC and DPPH• antioxidant activity under multiple storage conditions. Pearson correlation identified the relation between TPC and DPPH• antioxidant activity to be statistically significant (*p* < 0.05) at a value of 0.511, illustrating that the changes in TPC corresponded to the changes in the antioxidant activity during the entire stability study.

### 2.3. Impact of Storage Conditions on Individual Phenolic Compounds

GC/MS analysis was implemented to study the chemical composition in the dried extracts. This was achieved by studying the change of the individual phenolic compounds based on their corresponding peak area and expressed as a percentage of the total corrected peak areas. The results showed the presence of four major phenolic compounds, hydroxychavicol (66.5%), eugenol (12%), isoeugenol (3%) and 4-allyl-1,2-diacetoxybenzene (3.21%) in the dried *Piper betle* extracts. MANOVA statistics was implemented in the same manner as TPC and DPPH• antioxidant activity in order to determine the significance of each storage factor on the peak areas of all four phenolic compounds (Table S2).

The results revealed storage temperature had a statistically significant influence on the peak areas of hydroxychavicol (HC) (Table 3). Furthermore, it was the only compound that increased in its peak area, particularly for the extracts stored at 25 °C under light conditions by 20%, indicating possible oxidation of the compound. Auto-oxidation is common among phenolic compounds where they can be easily oxidised under the influence of light and temperature to form dimers [28]. Hydroxychavicol dimers have been previously identified in the ethanolic root extracts of *Piper betle* [29]. Statistically, neither light nor temperature was found to have any significant impact on eugenol (Table 4) as it maintained moderate stability under all conditions except for 25 °C in light. Isoeugenol, however, was heavily influenced by storage temperature (Table 5). More importantly, no isoeugenol was detected in the samples at 25 °C with or without the presence of light after 30 days of storage, indicating its massive decomposition at high temperature. A similar behavior was also expressed by 4-allyl-1,2-diacetoxybenzene (Table 6), which showed 100% degradation after the initial 15 days despite temperature and light.

Different phenolics behave in different manner when it comes to their degradation and this diverse behavior could be attributed to their structural difference. The reactivity of compound is dependent on the position of the functional groups. Positions 3 and 4 in the benzene ring of flavonoids are more susceptible to dihydroxylation than others [30]. Hydroxylation decreases the stability whereas methylation increases the stability of a compound [31]. As no detailed literature exists on the degradation mechanism of the phenolic compounds in *Piper betle* extracts, it is possible that similar hydroxylation of the hydroxyl group in 3 position of the benzene ring in eugenol and isoeugenol took place that led to its degradation. Similarly, it can only be assumed that the different structural variation including the number and position of diverse functional groups present in 4-allyl-1,2-diacetoxybenzene could have affected its reactivity and consequent degradation. 

In similar context, the structural difference could very well explain the good stability displayed by HC. Almost 100% of HC was retained under various storage conditions of temperature and light. Positions 1 and 2 hydroxyl groups in the benzene ring of HC might have contributed to its greater stability. This result was consistent with previous findings where 3-deoxyanthocyanins (3-DXAs) is more stable than other type of anthocyanins, due to its unique feature in its structure where there is a hydroxyl group lacking at C-3 position [32,33].

### 2.4. Detection of a Phenolic Compound during Stability Study

While observing the stability of the four major phenolic compounds in *Piper betle* extract, another phenolic compound was detected after 60 days of storage for samples exposed to light under all temperatures (Table 7). This phenol was identified through GC/MS as 2,4-bis-(1,1-dimethylethyl), commercially referred as 2,4-di-*tert*-butylphenol (2,4-DTBP). Moreover, it was found to gradually increase over time for both temperatures. 2,4-DTBP is an organic compound that is utilised in the industry as an intermediate for the production of ultraviolet (UV) stabilisers, and in the manufacturing of fragrances and pharmaceuticals. Typically, this compound is synthesised by the alkylation of a phenol with isobutylene in the presence of acid catalysts [34]. Specific precursors or the pathway for the production of this compound in *Piper betle* extract is yet to be fully identified. It was previously reported that the formation of 2,4-DTBP could be linked to the shikimate pathway [35]. The shikimate pathway is an alternative pathway for the biosynthesis of organic amino acids employed by plants and microorganisms [36,37]. It consists of seven enzymatic reactions before the final reductions of coniferyl alcohol to eugenol and isoeugenol [38]. Therefore, there may exist a possibility that 2,4-DTBP is related to the consequent degradation of eugenol, isoeugenol, or even both. 

The formation of 2,4-DTBP in the latter stages was a very noteworthy find. Some studies have reported that the degradation products have equal or in some cases better antioxidant potential than their predecessors [39,40]. The results corroborate with the present study as more than 90% of the antioxidant activity was retained under all storage conditions, despite degradation of some of the phenolic antioxidants (eugenol and isoeugenol). This result suggests that 2,4-DTBP may have contributed to the overall antioxidant activity. The antioxidant activity of 2,4-DTBP was studied in the previous findings by Choi et al. [35]. Varsha et al. reported 2,4-DTBP to be a potent antioxidant compound against amyloid-beta peptide-induced neurotoxicity. 2,4-DTBP also demonstrated a remarkable result of 80% total antioxidant capacity (TAC) and 33% ferric reducing power, which was highly comparable to that of ascorbic acid, with 83% TAC antioxidant capacity and 50% ferric reducing power respectively [41]. 

### 2.5. Degradation Kinetic Studies

Both zero-order and first-order kinetic models were implemented to investigate the degradation kinetics of dried *Piper betle* extract. The models were limited to antioxidant activity under the various conditions, as present study only aimed to investigate the stability of the antioxidant potential of the extracts. The experimental data fitting to the respective models are provided in Figures S1 and S2. The corresponding kinetic parameters from Table 8 clearly show that the degradation rate constants (*k*_1_ and *k*_2_) for both models were highest at 25 °C in the presence of light. Furthermore, *R*^2^ and root mean square error (*RMSE*) correlations determined indicates that the degradation kinetics of dried *Piper betle* extract were best described by zero-order kinetic model. Referring to literature, first-order kinetic model is the more common model of the two used in degradation studies [39,42]. However, present study found zero-order kinetic model to have an edge over first-order at high temperature. This could be because degradation of the antioxidants at 25 °C took place at a slower rate based on the zero-order kinetic model, instead of the rapid degradation as described by the first-order kinetic model [43]. Half-lives based on the zero-order kinetic model were determined to be 62 and 31 days for 5 °C, and 31 and 12 for 25 °C, under light and dark conditions respectively. 

## 3. Materials and Methods

### 3.1. Chemical and Reagents

Fast blue BB (FBBB) and DPPH• of analytical grade were purchased from Sigma Aldrich (Sigma Aldrich GmbH, Steinheim, Germany). 95% ethanol, 99.9% methanol and chloroform (high performance liquid chromatography grade), gallic acid standard and sodium hydroxide pellets were purchased from Sigma Aldrich (Sigma, St. Louis, MO, USA).

### 3.2. Plant Sample Preparation

Fresh *Piper betle* leaves were purchased from a local shop in Kuala Lumpur. Cleaned and cut leaves were air-dried at 50°C for a day using an air-forced convection oven (FAC-350, Protech, Sparks, NV, USA). 

### 3.3. Ultrasound-Assisted Extraction (UAE) of Bioactive Compounds from Piper betle

Prior to extraction, the dried samples were ground and sieved through an 800 µm sieve mesh to ensure the uniformity and symmetry of particle size. Ultrasound-assisted extraction was performed as outlined by Xu et al. [44]. The extraction parameters were based on previous optimisation studies which were identified at 52 °C with a solid to liquid ratio of 1:22 g/mL with 79% ethanol solution for a duration of 30 min [45]. Extraction was executed using an ultrasonic bath system (P120 H, Elmasonic, Singen, Germany) at a constant frequency and power of 37 kHz and 400 W respectively. Dried extract was obtained by evaporating the solvent at 50 °C for 30 min with a vacuum rotary evaporator (Hei-VAP Platinum 3, Heidolph, Schwabach, Germany). The dried extracts were stored in sealed containers under different storage conditions for thermal and photo-stability investigations.

### 3.4. Sample Storage Conditions

The storage conditions were based on World Health Organization (WHO) stability testing guidelines of active pharmaceutical ingredients and finished pharmaceutical products [46]. As present study only investigated the dried extract of *Piper betle* containing bioactive compounds with antioxidant properties, the stability guidelines for the active pharmaceutical ingredients were followed. Samples were stored in transparent bottles (with exposure to light) and amber bottles (to prevent the exposure to light) at 5 and 25 °C. Total phenolic content was quantified via FBBB. The chemical composition of the dried extracts was studied via GC/MS analysis. The corresponding antioxidant activity was determined with DPPH• radical scavenging assay. All subsequent analyses were executed every 15 days for a storage period of six months.

### 3.5. Total Phenolic Content

Total phenolic content (TPC) analysis of *Piper betle*’s extracts was determined according to Medina [47]. 0.1 mL of 0.1% of FBBB reagent was added to sample solutions of 0.05 mg/mL extracts in deionised water. The mixture was homogenised for a minute before the addition of 0.1 mL of 5% sodium hydroxide solution. Consequently, the mixtures were kept at room temperature (25 ± 2 °C) for 90 min for the reaction to reach completion. 200 µL of the sample mixtures were pipetted to a 96-well plate. The absorbance of the samples was analysed using a microplate spectrophotometer (Epoch 2, BioTek, Winooski, VT, USA) which was set at 420 nm. The retained TPC, obtained from triplicate analysis, was expressed in terms of mg gallic acid equivalent/g of dried extract according to the regression equation of gallic acid calibration curve obtained for the concertation range of 0 to 500 µg/mL (*R*^2^ = 0.9899). 

### 3.6. DPPH• Radical Scavenging Assay

A modified version of the DPPH• radical scavenging assay was followed, as described by Pin et al. [48]. 160 µL of 0.5 mg/mL extract solutions in 80% ethanol were transferred to a 96-well plate. This was followed by the addition of 40 µL of working 1 mM DPPH• methanolic solution. The plate was kept in the dark for 3 min in ambient temperature (25 ± 2 °C). The absorbance of the sample solutions were read at 520 nm via a microplate spectrophotometer. The radical scavenging activity, obtained from triplicate analysis, was expressed as % inhibition activity with the following Equation (2) [48]:

(1) $$DPPH\;\%\;Inhibition\;activity=\frac{A_{b}-A_{S}}{A_{b}}\times 100$$

where *A_b_* is the absorbance of blank solution containing DPPH• only and *A_s_* is the absorbance of the solution containing DPPH• with *Piper betle* extract.

### 3.7. Gas Chromatography/Mass Spectroscopy Assay

The peak areas of the individual phenolic compounds were monitored throughout the storage period using a GC/MS (7890A, Agilent Technologies, Petaling Jaya, Malaysia) analysis which was followed as elaborated by Foo et al. with slight modifications [49]. The initial oven temperature was set at 70 °C that was raised to 305 °C at a rate of 20 °C/min. The carrier gas (helium) was injected at a rate of 1.2 mL/min. The injector temperature and ion source temperatures were programmed at 250 °C and 230 °C respectively. 1 mL of 0.1 mg/mL extract solutions were injected into the capillary column in split ratio of 1:10 for run time of approximately 17 min. The individual compounds were identified using the compound’s mass spectrum with National Institute of Standards and Technology (NIST) Mass Spectral library (version 2).

### 3.8. Degradation Kinetic Studies

The degradation kinetics of antioxidant activity was evaluated using zero-order and first-order kinetic models in Equations (3) and (4) as described by Sapei and Hwa [43]:

(2) $$C_{t}=C_{o}-k_{1}t$$

(3) $$C_{t}=C_{o}\times exp^{-k_{2}t}$$

where *k*_1_ and *k*_2_ are the degradation rate constants, *C_o_* is the antioxidant activity when *t* = 0, and *C_t_* is the antioxidant activity at any given time. Half-life is the estimated time required for the antioxidant activity of the phenolic compounds to decrease by 50%. It was determined using Equation (5) as below which is based on the rate constant from the two kinetics models above that gave the best fit to the experimental data:

(4) $$t_{\frac{1}{2}}=-\frac{\ln0.5}{k}$$

### 3.9. Statistical Analysis

All of the results reported are an average of triplicate analysis ± standard deviation. Statistical analysis of MANOVA was performed to investigate the significance of light and temperature on total phenolic content and DPPH• antioxidant activity of the dried extracts (*p* < 0.05). Pearson’s correlation was performed to determine the correlation between TPC and DPPH• antioxidant activity. All of the statistical analysis was performed using IBM statistical package for the social sciences (SPSS) statistic software ver. 23 (IBM Corporation, Somers, NY, USA). Matlab 2017a (The MathWorks Inc., Natick, MA, USA) was used for model fitting of the experimental data to the kinetic models. Statistical correlation of *R*^2^ and *RMSE* were implemented for validation purposes.

## 4. Conclusions

Present study investigated the influence of multiple storage parameters, including temperature and light, on the stability of dried *Piper betle* extract. The dried extracts stored at low temperatures (5 °C) without exposure to natural light showed highest retention of phenolic content, with equally good corresponding DPPH• antioxidant activity after a storage time of 180 days. Hydroxychavicol displayed the best stability with 100% retention through the entire storage period. Eugenol and isoeugenol displayed moderate stability at low temperatures in dark conditions. 4-allyl-1,2-diacetoxybenzene suffered the worst with 100% degradation after 15 days at the set conditions. Despite the degradation of some of the phenolic compounds, more than 90% of the antioxidant activity was retained after 180 days under all conditions. A new compound, 2,4-di-*tert*-butylphenol, was detected after five weeks of storage, only in the extracts exposed to light. Zero-order kinetic models gave the best fit (*R*^2^ = 0.9046) to the degradation kinetics of DPPH• antioxidant activity. The overall quality of the dried extracts after six months was optimised at 5 °C in dark space. Half-life at the optimised storage condition from the zero-order kinetic model was determined as 62 days. It is suggested that the dried extracts of *Piper betle* should be stored at the optimum conditions as proposed in this study.

## Figures and Tables

**Table 1:** Stability of total phenolic content (mg gallic acid equivalent/g) of dried *Piper betle* extract under different storage temperatures and light for six months ^1^.

Temperature (°C)		Storage Time (Days)													% Retention
		0	15	30	45	60	75	90	105	120	135	150	165	180	
5	Light	311.28 ± 0.14	316.01 ± 0.25	312.98 ± 0.31	311.61 ± 0.37	312.35 ± 0.29	304.35 ± 0.27	314.82 ± 0.15	312.55 ± 0.31	313.45 ± 0.25	313.51 ± 0.14	314.38 ± 0.15	311.15 ± 0.20	310.95 ± 0.18	99.89
	Dark	311.28 ± 0.42	313.35 ± 0.32	320.65 ± 0.27	319.45 ± 0.15	315.78 ± 0.47	316.15 ± 0.37	315.35 ± 0.19	319.21 ± 0.36	320.41 ± 0.32	316.15 ± 0.09	316.21 ± 0.31	312.11 ± 0.36	311.51 ± 0.26	99.08
25	Light	311.28 ± 0.36	303.51 ± 0.26	302.71 ± 0.22	302.05 ± 0.24	305.71 ± 0.20	301.81 ± 0.42	310.81 ± 0.32	314.45 ± 0.22	295.45 ± 0.33	308.35 ± 0.26	306.01 ± 0.17	291.85 ± 0.15	289.63 ± 0.15	93.05
	Dark	311.28 ± 0.21	307.15 ± 0.46	303.28 ± 0.15	303.38 ± 0.17	302.51 ± 0.11	310.25 ± 0.26	306.78 ± 0.15	308.01 ± 0.24	304.78 ± 0.25	308.61 ± 0.24	305.88 ± 0.15	303.98 ± 0.20	302.43 ± 0.13	97.16

^1^ Values are expressed as mean ± standard deviation (SD) of triplicate experiments.

**Table 2:** Stability of 2,2-diphenyl-1-picrylhydrazyl (DPPH•) antioxidant activity (% inhibition) of dried *Piper betle* extracts under different storage temperatures and light for 6 months ^1^.

Temperature (°C)		Storage Time (Days)													% Retention
		0	15	30	45	60	75	90	105	120	135	150	165	180	
5	Light	95.79 ± 0.12	95.41 ± 0.08	94.56 ± 0.07	92.84 ± 0.13	93.64 ± 0.16	92.21 ± 0.13	93.41 ± 0.26	93.24 ± 0.15	91.47 ± 0.26	92.04 ± 0.23	91.55 ± 0.24	91.69 ± 0.16	91.59 ± 0.15	95.72
	Dark	95.79 ± 0.04	94.22 ± 0.13	94.13 ± 0.04	94.42 ± 0.16	94.42 ± 0.25	94.30 ± 0.17	94.39 ± 0.21	95.16 ± 0.13	95.32 ± 0.21	95.85 ± 0.18	96.40 ± 0.06	95.77 ± 0.12	95.72 ± 0.12	99.98
25	Light	95.79 ± 0.24	95.57 ± 0.24	94.27 ± 0.15	93.93 ± 0.05	93.19 ± 0.21	90.83 ± 0.05	93.65 ± 0.16	91.54 ± 0.07	90.28 ± 0.14	89.66 ± 0.15	86.26 ± 0.18	86.06 ± 0.15	86.12 ± 0.10	89.91
	Dark	95.79 ± 0.17	95.40 ± 0.14	94.56 ± 0.18	94.37 ± 0.22	92.89 ± 0.18	94.81 ± 0.19	91.24 ± 0.05	91.76 ± 0.15	92.62 ± 0.08	92.90 ± 0.15	92.08 ± 0.06	91.84 ± 0.25	91.64 ± 0.20	95.67

^1^ Values are expressed as mean ± SD of triplicate experiments.

**Table 3:** Stability profile of hydroxychavicol (HC) under different storage temperatures and light for 180 days ^1^.

Temperature (°C)		Storage Time (Days)													% Retention
		0	15	30	45	60	75	90	105	120	135	150	165	180	
5	Light	81.97 ± 1.12	85.41 ± 0.83	94.04 ± 0.48	94.02 ± 0.61	84.92 ± 0.46	85.57 ± 0.35	85.14 ± 0.95	86.42 ± 0.86	87.58 ± 0.35	88.57 ± 0.59	89.35 ± 0.75	89.32 ± 0.33	89.3 ± 0.37	108.94
	Dark	81.97 ± 0.62	83.40 ± 0.77	91.40 ± 0.66	92.31 ± 0.74	88.71 ± 0.84	90.02 ± 0.36	90.25 ± 0.37	91.97 ± 0.74	91.82 ± 0.73	91.56 ± 0.55	91.75 ± 0.36	91.81 ± 0.43	91.79 ± 0.33	111.98
25	Light	81.97 ± 0.83	85.44 ± 0.41	96.51 ± 0.42	96.87 ± 0.55	96.67 ± 0.37	92.75 ± 0.57	92.94 ± 0.41	95.81 ± 0.32	97.84 ± 0.70	97.94 ± 0.51	98.05 ± 0.64	97.94 ± 0.36	97.74 ± 0.93	119.24
	Dark	81.97 ± 0.51	86.65 ± 0.63	96.42 ± 0.53	96.47 ± 0.57	94.45 ± 0.42	94.28 ± 0.42	94.79 ± 0.79	95.92 ± 0.31	94.98 ± 0.85	95.53 ± 0.43	95.64 ± 0.76	95.63 ± 0.78	95.61 ± 0.85	116.64

^1^ Values are expressed as mean ± SD of triplicate experiments.

**Table 4:** Stability profile of eugenol under different storage temperatures and light for 180 days.

Temperature (°C)		Storage Time (Days)													% Retention
		0	15	30	45	60	75	90	105	120	135	150	165	180	
5	Light	11.92 ± 0.35	11.83 ± 0.85	3.60 ± 0.65	3.86 ± 0.71	5.45 ± 0.71	4.84 ± 0.74	4.71 ± 0.93	4.43 ± 0.36	4.86 ± 0.56	4.86 ± 0.49	5.27 ± 0.74	5.22 ± 0.52	5.224 ± 0.67	56.23
	Dark	11.92 ± 0.52	11.17 ± 0.44	4.14 ± 0.36	3.93 ± 0.35	4.32 ± 0.84	5.36 ± 0.62	5.25 ± 0.42	4.58 ± 0.35	5.24 ± 0.69	5.48 ± 0.44	5.30 ± 0.77	5.26 ± 0.41	5.22 ± 0.65	55.87
25	Light	11.92 ± 0.74	12.81 ± 0.37	3.49 ± 0.36	3.13 ± 0.57	3.33 ± 0.47	5.53 ± 0.85	5.22 ± 0.39	2.13 ± 0.52	0.00	0.00	0.00	0.00	0.00	0.00
	Dark	11.92 ± 0.35	11.83 ± 0.85	3.60 ± 0.65	3.86 ± 0.71	5.45 ± 0.71	4.84 ± 0.74	4.71 ± 0.93	4.43 ± 0.36	4.86 ± 0.56	4.86 ± 0.49	5.27 ± 0.74	5.22 ± 0.52	5.224 ± 0.67	63.31

**Table 5:** Stability profile of isoeugenol under different storage temperatures and light for 180 days.

Temperature (°C)		Storage Time (Days)													% Retention
		0	15	30	45	60	75	90	105	120	135	150	165	180	
5	Light	2.90 ± 0.45	2.76 ± 0.35	2.37 ± 0.62	2.12 ± 0.69	5.73 ± 0.67	4.28 ± 0.53	4.58 ± 0.74	3.41 ± 0.76	2.27 ± 0.64	1.31 ± 0.45	0.00	0.00	0.00	0.00
	Dark	2.90 ± 0.65	2.89 ± 0.55	2.75 ± 0.47	2.71 ± 0.74	5.95 ± 0.47	4.63 ± 0.73	4.51 ± 0.63	3.45 ± 0.78	2.94 ± 0.42	2.96 ± 0.53	2.96 ± 0.37	2.93 ± 0.59	2.932 ± 0.46	100.90
25	Light	2.90 ± 0.63	1.75 ± 0.48	0.00	0.00	0.00	0.00	0.00	0.00	0.00	0.00	0.00	0.00	0.00	0.00
	Dark	2.90 ± 0.57	1.89 ± 0.46	0.00	0.00	0.00	0.00	0.00	0.00	0.00	0.00	0.00	0.00	0.00	0.00

**Table 6:** Stability profile of 4-allyl-1,2-diacetoxybenzene under different storage temperatures and light for 180 days.

Temperature (°C)		Storage Time (Days)													% Retention
		0	15	30	45	60	75	90	105	120	135	150	165	180	
5	Light	3.21 ± 0.52	0.00	0.00	0.00	0.00	0.00	0.00	0.00	0.00	0.00	0.00	0.00	0.00	0.00
	Dark	3.21 ± 0.36	2.55 ± 0.47	1.71 ± 0.63	1.05 ± 0.65	1.01 ± 0.36	0.00	0.00	0.00	0.00	0.00	0.00	0.00	0.00	0.00
25	Light	3.21 ± 0.46	0.00	0.00	0.00	0.00	0.00	0.00	0.00	0.00	0.00	0.00	0.00	0.00	0.00
	Dark	3.21 ± 0.37	0.00	0.00	0.00	0.00	0.00	0.00	0.00	0.00	0.00	0.00	0.00	0.00	0.00

**Table 7:** Stability profile of 2,4-di-*tert*-butylphenol (2,4-DTBP) under different storage temperatures and light for 180 days.

Temperature (°C)		Storage Time (Days)													% Retention
		0	15	30	45	60	75	90	105	120	135	150	165	180	
5	Light	0.00	0.00	0.00	0.00	3.90 ± 0.47	5.32 ± 0.33	5.57 ± 0.55	5.74 ± 0.94	5.29 ± 0.47	5.27 ± 0.59	5.38 ± 0.67	5.46 ± 0.93	5.41 ± 0.95	38.68
	Dark	0.00	0.00	0.00	0.00	0.00	0.00	0.00	0.00	0.00	0.00	0.00	0.00	0.00	0.00
25	Light	0.00	0.00	0.00	0.00	0.00	1.72 ± 0.42	1.85 ± 0.36	2.06 ± 0.48	2.16 ± 0.48	2.06 ± 0.68	1.95 ± 0.35	2.06 ± 0.49	2.04 ± 0.53	18.81
	Dark	0.00	0.00	0.00	0.00	0.00	0.00	0.00	0.00	0.00	0.00	0.00	0.00	0.00	0.00

**Table 8:** Kinetic parameters of zero-order and first-order models.

Storage Temperature (°C)	Light	Zero-Order Kinetic Model				First-Order Kinetic Model		
		*k*_1_	*R*^2^	*RMSE*	*t*^1/2^	*K*_2_	*R*^2^	*RMSE*
5	Present	0.023	0.7895	0.710	30.77	−1.18 × 10^−4^	0.7908	2.89 × 10^−3^
5	Absent	−0.011	0.8545	0.275	61.78	2.41 × 10^−4^	0.8549	7.57 × 10^−3^
25	Present	0.057	0.9046	1.129	12.17	6.27 × 10^−4^	0.903	1.27 × 10^−3^
25	Absent	0.022	0.6873	0.913	31.24	2.37 × 10^−4^	0.6859	9.79 × 10^−3^

## Supplementary Materials

Table S1: MANOVA of TPC and DPPH antioxidant activity of dried *Piper betle*’s extracts, Table S2: MANOVA of individual phenolic compounds in dried *Piper betle*’s extracts, Figure S1: Zero-order kinetic model fitted to dried *Piper betle* extract’s antioxidant activity stored at (a) 5 °C and (b) 25 °C, Figure S2: First-order kinetic model fitted to dried *Piper betle* extract’s antioxidant activity stored at (a) 5 °C and (b) 25 °C.
