# Supplementary material for: Impact of Storage Conditions on the Stability of Predominant Phenolic Constituents and Antioxidant Activity of Dried Piper betle Extracts
Source: Molecules. 2018 Feb 23;23(2):484. doi: 10.3390/molecules23020484 (PMC6017861; doi:10.3390/molecules23020484)
Supplement: Supplementary file 1 [file molecules-23-00484-s001.docx]

Supplementary Data: Impact of Storage Conditions on the Stability of Predominant Phenolic Constituents and Antioxidant Activity of Dried
*Piper betle* Extracts

**Table S1.** MANOVA of TPC and DPPH antioxidant activity of dried *Piper betle’s* extracts*.*

| Independent variable | Dependent variable | Mean squares | *F*-value | Significance  (*p*) | Partial eta-squared |
| --- | --- | --- | --- | --- | --- |
| Light | TPC | 100.804 | 5.892 | 0.019 | 0.118 |
|  | DPPH antioxidant activity | 34.425 | 8.401 | 0.006 | 0.160 |
|  | TPC | 959.977 | 56.109 | 0.000 | 0.56 |
| Temperature | DPPH antioxidant activity | 26.746 | 6.526 | 0.014 | 0.129 |
| Light*Temperature | TPC | 13.825 | 0.808 | 0.374 | 0.018 |
|  | DPPH antioxidant activity | 0.100 | 0.024 | 0.877 | 0.001 |

**Table S2.** MANOVA of individual phenolic compounds in dried Piper betle’s extracts

| Independent variable | Dependent variable | Mean squares | *F*-value | Significance  (*p*) | Partial eta-squared |
| --- | --- | --- | --- | --- | --- |
| Light | Eugenol | 11.427 | 1.071 | 0.306 | 0.024 |
|  | Isoeugenol | 1.936 | 1.318 | 0.257 | 0.028 |
|  | Hydroxychavicol | 5.610 | 0.305 | 0.584 | 0.007 |
|  | Allylpyrocatechol 3,4-diacetate | 0.832 | 0.859 | 0.359 | 0.018 |
|  | 2,4 DTBP | 64.844 | 32.773 | 0.000 | 0.427 |
| Temperature | Eugenol | 13.399 | 1.255 | 0.269 | 0.028 |
|  | Isoeugenol | 84.058 | 57.255 | 0.000 | 0.565 |
|  | Hydroxychavicol | 316.367 | 17.202 | 0.000 | 0.281 |
|  | Allylpyrocatechol 3,4-diacetate | 0.832 | 0.859 | 0.359 | 0.019 |
|  | 2,4 DTBP | 16.415 | 8.269 | 0.006 | 0.159 |
| Light*Temperature | Eugenol | 8.350 | 0.782 | 0.381 | 0.017 |
|  | Isoeugenol | 1.825 | 1.243 | 0.271 | 0.027 |
|  | Hydroxychavicol | 21.894 | 1.190 | 0.281 | 0.026 |
|  | Allylpyrocatechol 3,4-diacetate | 0.832 | 0.859 | 0.359 | 0.019 |
|  | 2,4 DTBP | 16.415 | 8.269 | 0.006 | 0.159 |

(a)

(b)

**Figure S1.** Zero-order kinetic model fitted to dried *Piper betle* extract’s antioxidant activity stored at (a) 5 °C and (b) 25 °C

(a)

(b)

**Figure S2.** First-order kinetic model fitted to dried *Piper betle* extract’s antioxidant activity stored at (a) 5 °C and (b) 25 °C
